# A Rare Case of Ruptured Tailgut Cyst Leading to Carcinomatosis

**DOI:** 10.1155/2023/1282058

**Published:** 2023-05-03

**Authors:** Samir Atiya, Adam Horn, Whitney Wedel, Nicholas Lintel

**Affiliations:** ^1^Department of Pathology and Microbiology, University of Nebraska Medical Center, Omaha, Nebraska, USA; ^2^Department of Pathology, Mary Lanning Hospital, Hastings, Nebraska, USA

## Abstract

Tailgut cysts are congenital cysts arising in the retrorectal space. They are thought to be benign with variable malignancy risks. We report a case with previous surgical intervention decades prior that had undergone a tailgut cyst excision with surgical complications leading to carcinomatosis. An elderly female (70s) presented with tailbone/pelvic pain. She underwent cyst excision that was complicated by an intraoperative rupture. The cyst was pathologically proven to be a tailgut cyst with adenocarcinoma. She presented 13 months postoperatively to the emergency department with worsening abdominal pain. Imaging was concerning for diffuse omental nodules and narrowing of the proximal sigmoid colon. She was not deemed to be a surgical candidate and was transitioned to hospice care, where she passed away shortly afterward. This case report highlights the utility of complete excision of tailgut cysts and possible complications.

## 1. Introduction

Tailgut cysts (TGC) are congenital cysts arising in the retrorectal space from primitive tailgut remnants. They comprise only a small fraction of retrorectal tumors, and arise due to incomplete involution of the postcloacal gut. During embryogenesis, the embryo starts to fold inward during the 4th week. The lining can include a combination of epithelia, including transitional, glandular, and squamous.

Tailgut cysts have a female predominance of 5 : 1 [1] and typically occur in middle age, but can affect a wide age range [2]. Clinically, the findings range from an asymptomatic incidental finding to constipation, dyschezia, lower abdominal pain, abscess, and fever [3].

Previously, they were thought to behave in a benign fashion, with rare cases having cancer [4]. Lately, numerous papers have demonstrated that the malignant risk is higher than previous data, ranging from 8 to 26% [2, 5]. Of the malignant cases, adenocarcinomas and neuroendocrine tumors compose greater than 80%. To date, there have been less than 50 reported cases of tailgut cysts with adenocarcinoma [5–7] with a high rate of recurrence [7]. We report a rare case of an elderly female who initially had a pelvic mass that was removed and diagnosed as a tailgut cyst with adenocarcinoma that recurred a year later. Interestingly, she also had 2 previous operations decades prior (when she was 9 years old) for the removal of a “gelatinous mass.” To our knowledge, this is the first reported case of a tailgut cyst with an adenocarcinoma that had surgery decades prior in the same location. Furthermore, this case highlights the utility of complete excision to minimize recurrence.

## 2. Case Report

A 71-year-old well-nourished (BMI of 22.5) elderly patient presented to her physician for increasing pain in the tailbone/pelvic area for the past few months. She denied any complaints of bloody stool, changes in bowel movements, or urinary changes. She had not had a previous screening colonoscopy but has done yearly cologuard that has been negative. Given her clinical presentation, she underwent magnetic resonance imaging (MRI). The initial MRI demonstrated a large (10.6 × 12.8 × 14.1 cm) complex multiloculated cystic mass with solid components in the presacral space and extending into the perineum. T1 signaling demonstrated increased intensity, with findings likely secondary to intralesional hemorrhage or mucinous/proteinaceous components. Abdominal and pelvic computer tomography (CT) imaging performed at the same time as the MRI demonstrated a similarly large mass with multiloculated cysts. Within the septations, there were focal areas of calcification. On both imaging modalities, the mass is separate from the rectum and was suspicious for retrorectral cystic hamartoma, but the differential was broad. Given the mass effect and extension into the perineum, this most likely explains her presenting symptoms. Interestingly, the patient recalls a previous operation performed decades prior when she was approximately 9 years old to remove a cystic mass that was “gelatinous.” She did not recall any specific diagnosis or the specifics regarding the operation. Previous records were unretrievable due to the time elapsed between the two events, and previous healthcare providers were no longer reachable.

Given her history of a childhood mass decades prior, it was assumed that the mass was indolent in nature and not malignant. Plans to perform a colonoscopy were arranged with the goal of surgical excision. During surgery, the patient was placed in a supine position with careful preparation of the abdomen. A deep midline incision was performed with cautery down to the fascia after which the perineum was opened. The bowel was retracted, and a Balfour retractor was placed. Attention was focused on the perineum, where manual dissection was performed to free most of the mass. However, the portion of the mass within the pelvis was tightly adhered to the pelvis, coccyx, sigmoid colon, and rectum. With retractors and careful dissection, the mass was finally separated from important structures. When trying to skeletonize and shell out the mass, there was limited space for the retractor due to the size and firmness of the mass. An incision on the top aspect of the mass was performed where 800 cc of brownish fluid was suctioned with some spillage into the abdominal cavity. After the mass was partially decompressed, the anterior aspect was grasped with an Allis clamp and retracted anteriorly. Attention was then turned to the sacrum to remove the mass. The sacral vein unfortunately bled, leading to technical complications that were ultimately resolved with suture ligature and pressure. Unfortunately, the mass was severely adhered to the coccyx, and ultimately a complete excision was not possible (80% was removed). The pelvis was then washed with multiple liters of saline to ensure hemostasis was maintained as well as pelvic and abdominal cleaning. A 19-French round Blake drain was placed in the pelvis. The midline fascial wound was then closed, and the skin was stapled. Overall, the estimated blood loss was 2,500 milliliters.

**Figure 1:**
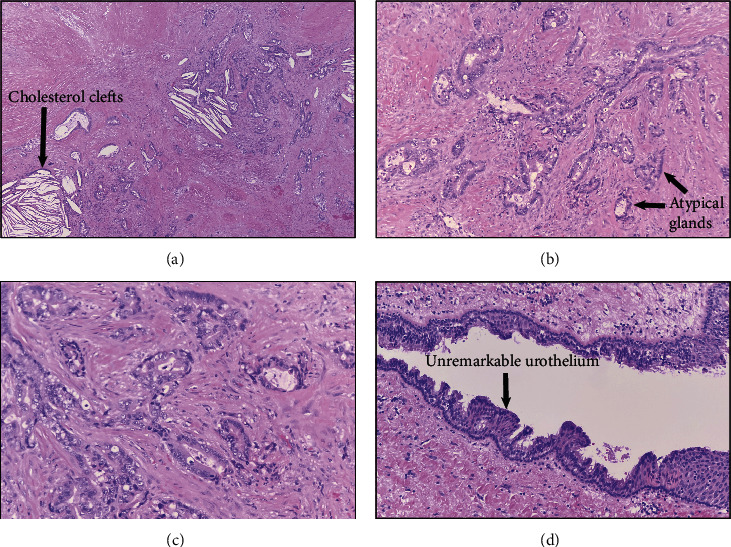
Hematoxylin and eosin (H&E) stain. Histology demonstrates fibrous tissue with cholesterol clefts and foamy histiocytes (a). Within the fibrous stroma, there are atypical cells within the stroma with atypical cells (b). On higher power (20x), the ducts are lined by moderately atypical cells (c). There are other areas of the normal transitional and columnar epithelium (d).

**Figure 2:**
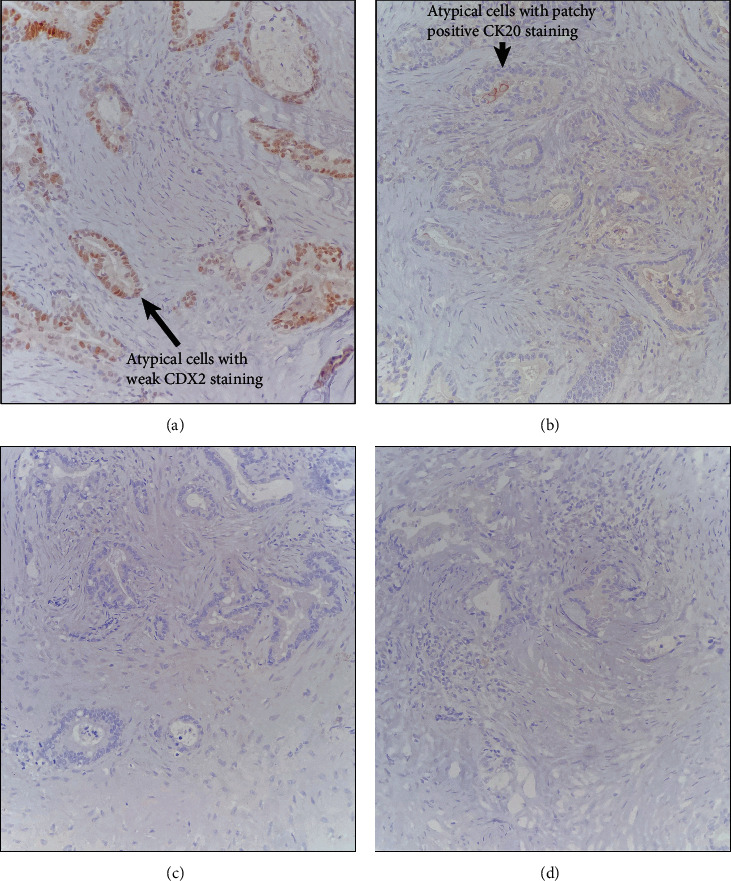
Immunohistochemical stains. The atypical areas stain positive for CDX2 (a) and patchy positive for CK20 (b). The tumor cells are negative for CK7 (c) and synaptophysin (d).

Histologically, the specimen demonstrated fibrous tissue with cholesterol clefts, foamy histocytes, necrosis, and focal calcification (Figure 1(a)). There were atypical glands with a background of desmoplastic stroma (Figure 1(b)). The cells had moderate cytologic atypia (Figure 1(c)) with discrete areas of the normal transitional and columnar epithelium (Figure 1(d)). Immunohistochemical staining demonstrated patchy staining for caudal-type homeobox 2 (CDX2) (Figure 2(a)) and weak staining for cytokeratin (CK) 20 (Figure 2(b)). CK7 (Figure 2(c)), paired-box gene 8 (PAX-8), synaptophysin (Figure 2(d)), chromogranin, and estrogen receptor (ER) were negative. The case was diagnosed as adenocarcinoma with the findings favored to represent the carcinoma arising from within a tailgut cyst.

She was then treated with FOLFOX followed by chemoradiation with continuous 5-fluorouracil for a 6-month duration of adjuvant treatment. 13 months postsurgery, she presented to the emergency department with worsening abdominal pain due to refractory constipation. A CT abdomen and pelvis were performed and demonstrated diffuse, ill-defined soft tissue density and areas of multiloculated fluid with omental nodules highly suspicious for carcinomatosis as well as narrowing of the proximal sigmoid colon. The surgical care team was consulted, and given her clinical presentation, she was not deemed a surgical candidate. After a discussion with the patient and the family, she was placed in hospice. However, she developed an ileus/obstruction with emesis refractory to nonoperative management. The decision was made to perform an exploration and create a diverting ostomy with the potential for a sigmoid colectomy depending on the location of the obstruction. Ultimately, an area of obstruction was found in the sigmoid colon, and resection was performed. Unfortunately, the patient succumbed to her disease and dies shortly after.

A gross examination of the resection showed the mucosa was mottled with patchy areas of necrosis but no areas of induration, masses, or polyps. Cut surfaces of the colon show multiple scattered ill-defined areas of white-tan induration in the colon wall and pericolonic tissue.

**Figure 3:**
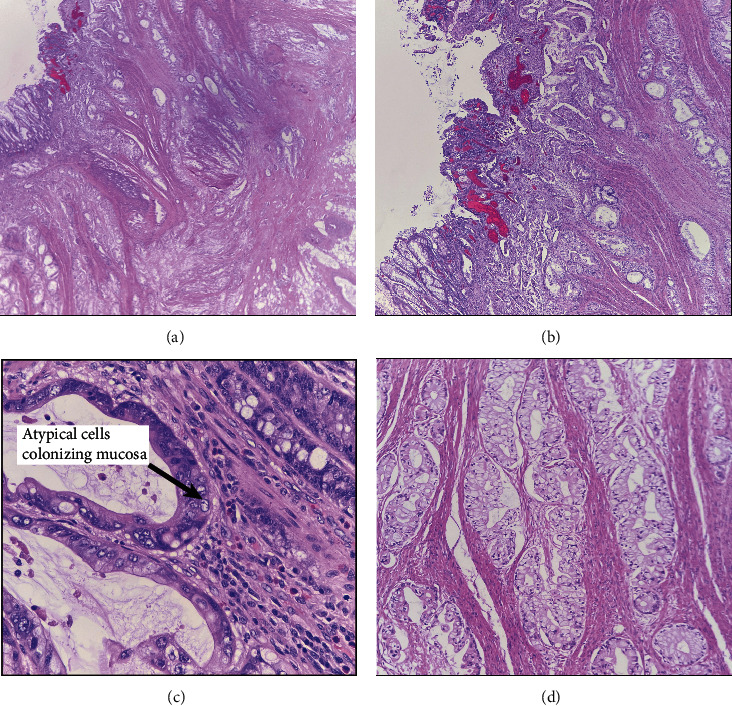
Hematoxylin and eosin (H&E) stain. Histology demonstrates a pyramidal-shaped tumor involving the overlying mucosa (a, b). Higher magnification (40x) demonstrates atypical tumor cells colonizing the overlying mucosa (c). Some areas demonstrate voluminous intracytoplasmic mucin (d).

**Figure 4:**
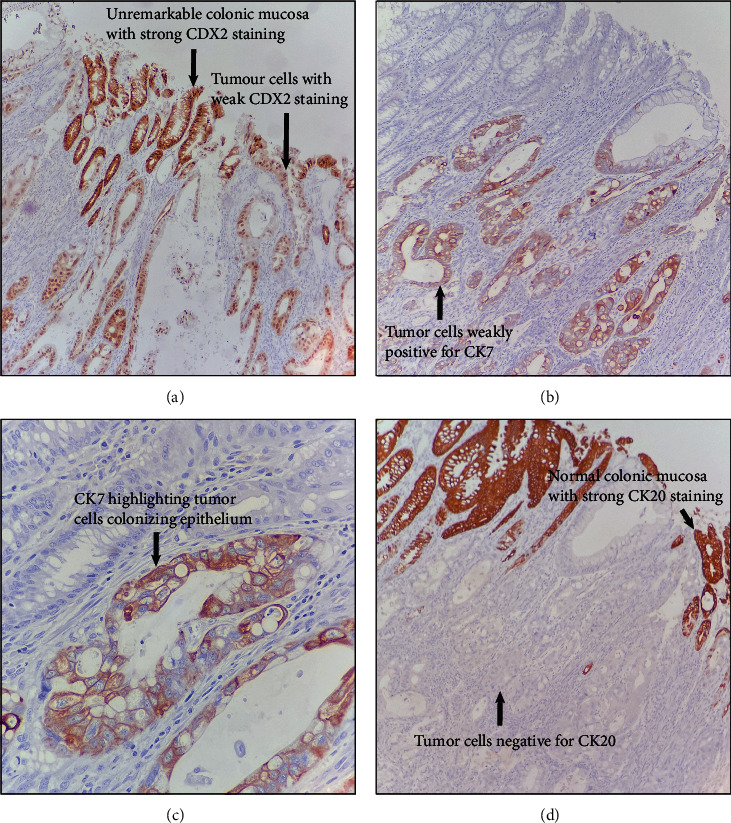
Immunohistochemical stains. The tumor stains weakly positive for CDX2 (a) and CK7 (b). CK7 also highlights the atypical tumor cells colonizing the overlying mucosa (c). CK20 is negative within the tumor but strongly positive in the surrounding unremarkable colonic mucosa (d).

Histological examination demonstrated similar morphology to the initial specimen but more patchy areas with voluminous intracytoplasmic mucin (Figures 3(a) and 3(d)). The bulk of the tumor was in the colon wall in a pyramid shape, with the apex of the tumor appearing to colonize the mucosa (Figure 3(b)). The atypical cells can be seen colonizing the colonic mucosa (Figure 3(c)). Immunohistochemistry demonstrated a tumor that is positive for CDX2 (weak) (Figure 4(a)) and CK7 (Figures 4(b) and 4(c)) while CK20 (Figure 4(d)) and PAX8 are negative. The surrounding unremarkable colonic tissue is strongly positive for CK20 (Figure 4(d)) and CDX2 (Figure 4(a)). The case was diagnosed as adenocarcinoma with a description of the findings and suggests that the tumor found is related to the prior specimen. The mild change in the expression of CK20 and CK7 was attributed to either tumor heterogeneity or provoked due to chemotherapy.

## 3. Discussion

Tumors occurring in the presacral space are extremely rare and estimated to affect less than a fraction of 1% [8]. However, given the complex ontogenesis of the region, numerous conditions can arise from this area including inflammatory (fistula, abscess, and granuloma), as well as neoplastic. Within the neoplastic category, the differential is wide and includes osseous mesenchymal tumors (osteoma, osteosarcoma, and Ewing sarcoma), mesenchymal soft tissue (liposarcoma, lipoma, fibrosarcoma, leiomyoma, and leiomyosarcoma), and neurogenic (neurofibroma, neurofibrosarcoma, and ganglioneuroma). Congenital origin (teratoma, tailgut cyst, and rectal duplication cyst) accounts for more than half of cases [8]. Of these, developmental cysts account for more than half [8]. However, TGC is rare congenital cysts derived from primitive tailgut remnants [1].

Tailgut cysts tend to occur in women with a ratio of 5 : 1 compared to men and have a wide age range [1, 8]. Half of all lesions are found incidentally [1, 8]. There is disagreement regarding the effectiveness of digital rectal exams [1, 8]. We believe the variability in the literature is due to the heterogeneous nature of the cyst. This further undermines the utility of radiological imaging. While some studies discuss hypointense T1 and hyperintense T2 [9, 10], other studies demonstrate a wide range of variability (especially on T1 imaging) [5, 11]. Given the broad differential, many etiologies can present with similar radiological findings, leading to more than 50% of cases being missed initially [2].

The original specimen was diagnosed as TGC with a carcinomatous portion arising within. There was weak CK20 and patchy staining for CDX2 which further add doubt to colorectal as the primary source. Additional immunohistochemical staining to rule out common sites of adenocarcinoma from the thyroid and Mullerian derived (PAX8), pancreatobiliary (CK7), breast (ER), and neuroendocrine tumors (synaptophysin and chromogranin) were all negative. When she presented the second time, there was discussion clinically that this might represent a colorectal primary. However, the gross findings did not suggest a colon primary as the mass was predominately submucosal with the apex abutting the colonic epithelium. Furthermore, the histology was similar to that of the primary specimen, and the immunostaining of the lesion is closer to that of the primary lesion while not having the stereotypical immunophenotype of a colonic adenocarcinoma (strong CK20 and CDX2 positivity).

The unique features of this case are the appearance of potential mucosal colonization of the tumor and the patient's history of prior surgery in the same anatomical location. Although efforts were made to find the records of the surgeries in her childhood, they could not be located. We speculate that the excision during her childhood could have been an incompletely excised tailgut cyst, given her age at the time. Over the decades, it could have enlarged and developed the carcinoma. The rupture during surgery, not an uncommon occurrence of these lesions, likely led to carcinomatosis and the findings in the colon wall.

## 4. Conclusion

We report a case of a tailgut cyst that was ruptured on initial excision and presented 13 months later with metastasis. Although tailgut cysts are rare, they should still be in the differential diagnosis of presacral lesions. Careful surgical excision is warranted to minimize the risk of recurrence and potential metastasis.

## References

[1] Haydar M., Griepentrog K. (2015). Tailgut cyst: a case report and literature review. *International Journal of Surgery Case Reports*.

[2] Broccard S. P., Colibaseanu D. T., Behm K. T. (2022). Risk of malignancy and outcomes of surgically resected presacral tailgut cysts: a current review of the Mayo Clinic experience. *Colorectal Disease*.

[3] Liang F., Li J., Yu K., Zhang K., Liu T., Li J. (2020). Tailgut cysts with malignant transformation: features, diagnosis, and treatment. *Medical Science Monitor: International Medical Journal of Experimental and Clinical Research*.

[4] Hjermstad B. M., Helwig E. B. (1988). Tailgut cysts. Report of 53 cases. *American Journal of Clinical Pathology*.

[5] Nicoll K., Bartrop C., Walsh S. (2019). Malignant transformation of tailgut cysts is significantly higher than previously reported: systematic review of cases in the literature. *Colorectal Disease*.

[6] Martins P., Canotilho R., Peyroteo M., Afonso M., Moreira A., Sousa A. (2019). Tailgut cyst adenocarcinoma. *Autopsy and Case Reports*.

[7] Kaistha S., Gangavatiker R., Harsoda R., Kinra P. (2018). A case of adenocarcinoma in a tail gut cyst and review of literature. *Medical Journal, Armed Forces India*.

[8] Akbulut S. (2013). Unusual cause of defecation disturbance: a presacral tailgut cyst. *European Review for Medical and Pharmacological Sciences*.

[9] Aflalo-Hazan V., Rousset P., Mourra N., Lewin M., Azizi L., Hoeffel C. (2008). Tailgut cysts: MRI findings. *European Radiology*.

[10] Al-Dasuqi K., Irshaid L., Mathur M. (2020). Radiologic-pathologic correlation of primary retroperitoneal neoplasms. *Radiographics*.

[11] Saba L., Fellini F., Greco F. G. (2014). MRI evaluation of not complicated tailgut cyst: case report. *International Journal of Surgery Case Reports*.

